# First-line axitinib therapy is less effective in metastatic renal cell carcinoma with spindle histology

**DOI:** 10.1038/s41598-020-77135-6

**Published:** 2020-11-18

**Authors:** Kazuyuki Numakura, Mizuki Kobayashi, Yumina Muto, Yuya Sekine, Makoto Takahashi, Soki Kashima, Ryohei Yamamoto, Atsushi Koizumi, Taketoshi Nara, Mitsuru Saito, Shintaro Narita, Hiroshi Nanjyo, Tomonori Habuchi

**Affiliations:** 1 Department of Urology, Akita University Graduate School of Medicine, 1-1-1 Hondo, Akita, 010-8543 Japan; 2 Division of Pathology, Akita University Hospital, Akita, Japan

**Keywords:** Renal cell carcinoma, Tumour biomarkers

## Abstract

Axitinib, a vascular endothelial growth factor receptor-tyrosine kinase inhibitor, will be used in combination first-line therapies against metastatic renal cell carcinoma (mRCC), but its effects as a first-line monotherapy are unclear. Thus, we aimed to elucidate pretreatment clinical factors that predict the prognosis of patients with mRCC receiving first-line axitinib therapy. We enrolled 63 patients with mRCC treated with axitinib as first-line therapy between Nov. 2003 and Jul. 2018. Progression-free survival (PFS) and overall survival (OS) were assessed using the Wald χ^2^ statistic in Cox proportional hazards regression. Median patient age was 67 (range: 25–85) years. Seven (11.1%) patients were classified as being at favorable risk, 33 (52.4%) at intermediate risk, and 23 (36.5%) at poor risk according to the International Metastatic Renal Cell Carcinoma Database Consortium (IMDC) risk classification system. Median follow-up duration after axitinib initiation was 14 (range: 1–72) months. Median PFS and OS were 18 months and 65 months, respectively. Cox regression analyses of clinical predictors revealed that high C-reactive protein (CRP) levels were significantly correlated with shorter PFS [hazard ratio (HR), 1.63; 95% confidence interval (CI) 1.7–4.0)], whereas spindle cells and poor IMDC risk scores were related to worse OS (HR, 2.87 and 2.88, respectively; 95% CI 1.4–11.0 and 1.1–8.5, respectively). Thus, patients with mRCC and spindle histology or poor IMDC risk scores had worse OS, and those with high CRP levels had shorter PFS in first-line axitinib treatment. Other therapies might be more suitable for initial management of such patients.

## Introduction

An advancement in combination therapy using immune checkpoint inhibitors (ICI) with axitinib, which is a vascular endothelial growth factor receptor (VEGFR)-tyrosine kinase inhibitor (TKI), or anti-cytotoxic T-lymphocyte associated antigen-4 (CTLA-4) antibody would provide a standard first-line therapy in treatment of metastatic renal cell carcinoma (mRCC)^1–3^. However, immune combination therapy has not shown a clear advantage over VEGFR-TKI therapy in patients with mRCC that have favorable International Metastatic Renal Cell Carcinoma Database Consortium (IMDC) risk scores^1–3^. In other words, certain patients are good candidates for first-line VEGFR-TKI monotherapy^4,5^. Additionally, since sunitinib has been the only VEGFR-TKI used as a control drug in these pivotal combination therapy studies, the clinical effects of other VEGFR-TKIs have not been fully evaluated yet in this setting^1–3^.

Axitinib provides higher selective inhibition of VEGFR 1–3 than do other VEGFR-TKIs and is approved as a second-line therapy for mRCC. This distinctive characteristic might allow for the same or better clinical outcomes as well as effective first-line therapy, although axitinib has not demonstrated improved prognosis over that of sorafenib in first-line settings^6^. In contrast, axitinib has resulted in superior clinical outcomes, in terms of both survival and safety profile, compared to those of sunitinib in a real-world retrospective cohort^7^.

Two new combination therapies consist of axitinib and ICIs. The combination is expected to provide a synergistic effect^8,9^, but information on outcomes of first-line axitinib monotherapy against mRCC in the subset of patients with favorable or intermediate IMDC risk scores is still needed.

The objective of this study was to elucidate pretreatment clinical factors that predict the prognosis of patients with mRCC receiving first-line axitinib therapy. Our results may improve the selection of candidates for combination therapies.

## Methods

### Eligibility criteria

Patients with histologically proven mRCC, regardless of Eastern Cooperative Oncology Group (ECOG) performance status (PS), were included in this study. One hundred and sixty patients were treated with VEGFR-TKI at the Akita University Hospital (Akita, Japan) between Nov. 2003 and Jul. 2018. This study enrolled 63 of these patients who were treated with axitinib as first-line therapy. Patients who were given axitinib as a presurgical treatment were excluded. This study was approved by our institutional review board (No. 2265). All procedures were performed in accordance with the ethical standards laid down in the 1964 Declaration of Helsinki.

### Primary and secondary endpoints

The primary objective was to assess clinical outcomes of axitinib treatment, including the objective response rate (ORR), progression-free survival (PFS) rate, overall survival (OS) rate, and incidence of adverse events (AEs). The secondary objectives were to identify pretreatment clinical factors that could affect the prognosis of patients with mRCC treated with first-line axitinib.

### Treatment and follow-up examinations

The following patient characteristics were recorded and laboratory tests were conducted before starting treatment and during therapy at the attending physician’s discretion: complete medical history, physical examination, ECOG PS, blood cell counts with differential and platelet counts, biochemical profile (including electrolytes, renal and hepatic function, coagulation, pancreatic amylase, and lipase), urinalyses, and chest radiography. Toxicity was graded using the Common Terminology Criteria for Adverse Events version 4.0. The size of each tumor was evaluated and measured using computed tomography scans obtained within 4 weeks before starting axitinib. After axitinib treatment was started, the assessment interval was scheduled for individual patients by the attending physicians. Tumor response was evaluated using the Response Evaluation Criteria in Solid Tumors guidelines version 1.1.

### Statistical analysis

PFS was defined as the time between the initiation of axitinib treatment and disease progression or death as confirmed by radiological images or obvious clinical manifestation of progressive disease. OS was defined as the time between axitinib initiation and death. The database record was closed upon patient death or the final follow-up. The cut-off value for neutrophil–lymphocyte ratio (NLR), serum albumin level, and C-reactive protein (CRP) level was set as their median values. Data are expressed as means ± standard deviation values, and differences with *p*-values less than 0.05 were considered statistically significant. The chi-squared test was used to examine differences in categorical data. PFS and OS were stratified using the Kaplan–Meier method. The Cox proportional hazard regression model was used for the analysis of hazard ratio (HR) and 95% confidence interval (CI). Analyses were performed using SPSS version 24.0 statistical software (SPSS, Inc., Chicago, IL, USA).

### Ethics approval

This study was approved by the institutional review board. All procedures were performed in accordance with the ethical standards laid down in the 1964 Declaration of Helsinki.

### Consent to participate

Informed consent was obtained from all individual participants included in the study.

### Consent for publication

Patients signed informed consent regarding publication of their data.

## Results

### Patient characteristics

The 63 enrolled patients received at least 2 weeks of axitinib therapy and were assessed for treatment efficacy and toxicity. The median patient age was 67 (range: 25–85) years. All patients were Japanese, and the cohort included 47 (74.6%) men and 16 (25.4%) women. Fifty (79.4%) patients underwent radical nephrectomy before starting axitinib therapy. Fifty-eight (92.1%) patients had clear cell histology, four (6.3%) had papillary, two each (3.0%) had chromophobe and Xp11.2 translocation, and thirteen (20.6%) had spindle histology. Thirty (47.6%) patients had an ECOG PS of 0, 13 (20.6%) had an ECOG PS of 1, and 11 (17.5%) had an ECOG PS of 2 or higher. Under the IMDC risk classification system, 7 (11.1%) patients were classified as being at favorable risk, 33 (52.4%) at intermediate risk, and 23 (36.5%) at poor risk. Eighteen (28.6%) patients had one metastatic site, 19 (30.2%) had two, and 26 (41.3%) had three or more (Table 1).

**Table 1:** Baseline demographic and clinical characteristics.

Total patient number	*N* = 63
**Age**	
Median year (range)	67 (25–85)
**Gender, n (%)**	
Male	47 (74.6)
Female	16 (25.4)
**Nephrectomy, n (%)**	
Yes	50 (79.4)
**Histology, n (%)**	
Clear cell	58 (92.1)
With spindle component	13 (20.6)
Papillary	4 (6.3)
Chromphobe	2 (3.2)
Xp11.2 translocation	2 (3.2)
**Clinical stage at the time of diagnosis with RCC, n (%)**	
1	17 (27.0)
2	6 (9.5)
3	8 (12.7)
4	32 (50.8)
**IMDC risk classification, n (%)**	
Favorable	7 (11.1)
Intermediate	33 (52.4)
Poor	23 (36.5)
**Metastatic site, n (%)**	
1	18 (28.6)
2	19 (30.2)
3	9 (14.3)
4 ≤	17 (27.0)
**ECOG PS, n (%)**	
0	39 (61.9)
1	13 (20.6)
2 ≤	11 (17.5)
**Site of metastases, n (%)**	
Lung	49 (77.8)
Lymph node	37 (58.7)
Bone	21 (33.3)
Liver	12 (19.0)
Adrenal gland	6 (9.5)
Brain	5 (7.9)
Opposite kidney	5 (7.9)
Others	11 (17.5)
**Time from diagnosis to systemic therapy, n (%)**	
< 1 year	31 (49.2)
**Neutrophil–lymphocyte ration, n (%)**	
> 2.9	31 (49.2)
**Hemoglobin, n (%)**	
< Lower limit of normal	49 (77.8)
**Platelet count, n (%)**	
> 34.8 × 10^4^/μL	11 (17.5)
**Albumin, n (%)**	
< 3.7 g/dL	30 (47.6)
**Corrected carcium, n (%)**	
> 10 mg/dL	4 (6.3)
**LDH, n (%)**	
> 1.5 × upper limit of normal	5 (7.9)
**CRP, n (%)**	
> 0.5 mg/dL	32 (50.8)

*IMDC* International Metastatic Renal Cell Carcinoma Database Consortium, *ECOG* Eastern Cooperative Oncology, *PS* performa status, *LDH* lactate dehydrogenase, *CRP* C-reactive protein.

### Antitumor effect

**Figure 1:**
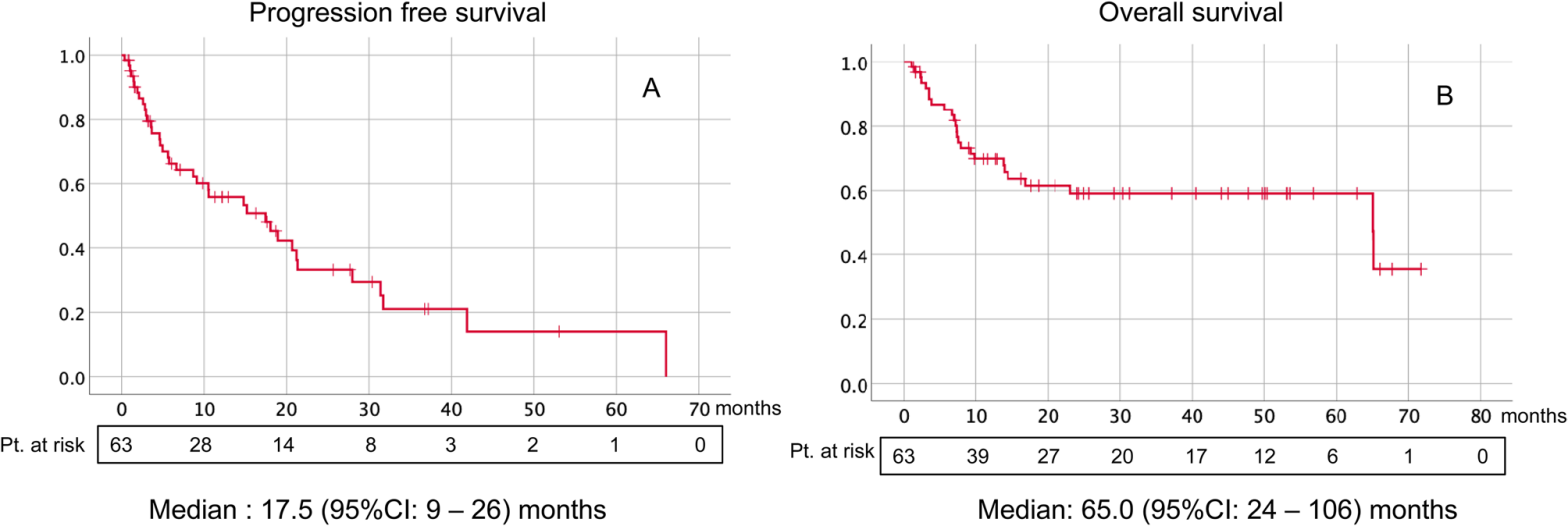
Kaplan–Meier curves of progression-free survival (**A**) and overall survival (**B**) for the entire study population (N = 63).

At the time of analysis, 12 patients (19.0%) were still being treated. The reasons for treatment discontinuation were progressive disease (58.7%) and AE (19.0%). An objective response was found in 25 (39.7%) patients (Table 2). The median PFS was 17.5 months (95% CI 9–26), and the overall survival was 65.0 months (95% CI 24–106) (Fig. 1). These survival rates are comparable to the median PFS (12.2 months) and OS (33.0 months) in the Japanese population from a randomized phase III trial^10^.

**Table 2:** Results of axitinib management.

**Initial dose (mg/day)**	
Median (range)	10 (6–10)
**Duration (month)**	
Median month (range)	9 (1–67)
**Current treatment status, n (%)**	
Continue	12 (19.0)
Discontinuation	51 (81.0)
**Cause of discontinuation**	
PD	37 (58.7)
AE	12 (19.0)
Others	2 (3.2)
**Objective response rate, n (%)**	
CR + PR	25 (39.7)
**Best response, n (%)**	
CR	2 (3.2)
PR	23 (36.5)
SD	23 (36.5)
PD	12 (19.0)
Not assessed	3 (4.8)

*PD* progressive disease, *AE* adverse event, *CR* complete response, *PR* partial response, *SD* stabel disease.

### Adverse events

By the time of the data cut-off, 29 patients (46.0%) had experienced an AE (Table 3), and 12 (19.0%) had discontinued nivolumab treatment because of AEs. The most common AEs of grade 3 or higher included 3 cases each of anorexia, general fatigue, hypertension, and proteinuria; two cases each of myocardial infarction, liver dysfunction, diarrhea, and pneumonia; and one case each of renal sufficiency, glossitis, shingles, aspiration pneumonia, and artery mural thrombosis (Table 3). There was no relationship between AE development and clinical response (data not shown).

**Table 3:** Treatment-related adverse events.

Total patient number	*N* = 63
Number of patients with AEs (any grade), n (%)	29 (45.5)
**Cause of discontinuation, n (%)**	
PD	37 (58.7)
AE	12 (19.0)
**The detail of AEs (G3 or more), n (%)**	15 (23.8)
Anorexia	3 (4.8)
Myocardial infarction	2 (3.2)
Liver dysfunction	2 (3.2)
Diarrhea	2 (3.2)
Pneumonia	2 (3.2)
Renal insufficiency	1 (1.6)
Proteinuria	1 (1.6)
Glossitis	1 (1.6)
Shingles	1 (1.6)
Aspiration pneumonia	1 (1.6)
Artery mural thrombus	1 (1.6)

*PD* progressive disease, *AE* adverse event, *G* grade.

### Multivariate analysis

The following 6 variables exhibited significance in univariate selective analysis of clinically relevant factors (Table 4) and were included in the multivariate model: poor IMDC risk score, not having undergone nephrectomy, spindle histology, NLR of 2.9 or more, CRP level of 0.5 or more, and 4 or more metastatic organs (Table S1 in the Supplementary Appendix). Cox regression analyses for clinical predictors revealed that high CRP value was significantly correlated with shorter PFS (HR, 1.63; 95% CI 1.7–4.0), whereas spindle histology and poor IMDC risk score were correlated with worse OS (HR, 2.87 and 2.88, respectively; 95% CI 1.4–11.0 and 1.1–8.5, respectively) (Table 4).

**Table 4:** Prognostic values of clinical variables for predicting progression-free survival and overall survival in 63 patients with metastatic renal cell carcinoma analyzed by using univariate and multivariate Cox proportional hazards regression models.

Risk factor	Risk category	Univariate				Multivariate					
		PFS		OS		PFS			OS		
		HR	p	HR	p	HR	95% CI	p	HR	95% CI	p
**IMDC risk classification**	**Poor**	**4.53**	**0.033**	**14.28**	** < 0.001**	1.72	–	0.189	**2.88**	**1.10–8.47**	**0.030**
Nephrectomy	Not performed	1.51	0.219	**6.22**	**0.013**				0.24	–	0.622
**Spindle histology**	**Positive**	**5.16**	**0.023**	**10.71**	**0.001**	1.96	–	0.162	**2.87**	**1.40–11.00**	**0.020**
NLR	2.9 or more	3.51	0.061	**8.72**	**0.003**				1.83	–	0.176
CRP	0.5 or more	**4.87**	**0.027**	**12.50**	**0.002**	**1.63**	**1.70–4.02**	**0.031**	3.07	0.97–7.75	0.058
Metastatic organ	4 or more	**5.22**	**0.022**	**7.30**	**0.007**	2.21	–	0.137	1.20	–	0.274

Statistically significant factors are shown in bold.

*PFS* progression-free survival, *OS* overall survival, *HR* Hazard ratio, *CI* confidence interval, *IMDC* International metastatic renal cell carcinoma database consortium, *NLR* neutrophil lymphocyte ratio, *CRP* C reactive protein.

## Discussion

This study focused on patients that were treated with axitinib as a first-line therapy and as a representative the VEGFR-TKI regimen. The clinical outcomes of these patients were reported, and prognostic factors for these patients were elucidated. The median OS of all patients was 65.0 months, which was comparable with the findings of subgroup analysis in a pivotal study on sunitinib^10^ and a first-line axitinib regimen^7^ in Japanese patients with mRCC. In addition, we identified CRP expression level as a potential prognostic factors of PFS and poor IMDC risk score and spindle histology as potential prognostic factors of OS. Patients that did not exhibit any of these adverse prognostic factors received long-term benefits from axitinib as a first-line treatment and showed better OS. These findings indicate that axitinib is effective and well-tolerated as a first-line treatment among patients with mRCC.

The results of this study were comparable to those of previous studies on first-line VEGFR-TKIs^11,12^. In our study and in a phase II trial of sunitinib, the median OS rates were 65.0 months and 33.1 months, respectively; the median PFS rates were 17.5 months and 12.2 months, respectively; and the ORRs were 39.7% and 52.0%, respectively^10^. The median duration of axitinib treatment was longer (9.0 months) in our study than in the phase II trial of sunitinib (6.0 months). The median OS in our study was better than those of other retrospective cohort studies (median OS: 65.0 to 33.2 months). The most common AEs of grade 3 or more that led to treatment discontinuation were anorexia, general fatigue, hypertension, and proteinuria. Although the most common AE (of any grade) in the first-line axitinib phase III trial (AXIS trial) was hypertension^13^, our treatment was not interrupted due to hypertension.

Two prognostic factors of OS were identified in our study. Poor IMDC risk score^14,15^ and spindle histology^16^, which have already been identified as markers of poor prognosis in second-line axitinib treatment cohorts, were shown to also be prognostic markers in the first-line axitinib setting. Although VEGFR-TKI monotherapy is still considered usable in all IMDC risk categories by both the European Association of Urology and the National Comprehensive Cancer Network treatment guidelines for mRCC^17,18^, the efficacy of VEGFR-TKIs does not satisfy us. In clinical practice, ICI combination therapy would be a better choice^19^, regardless of its complicating immune-related adverse events^20^. Moreover, since patients with mRCC and spindle histology received only a small benefit to survival in our study, ICI combination therapy seems a better strategy^21,22^. Specifically, combination therapy consisting of anti-CTLA-4 (ipilimumab) and anti-PD-1 (nivolumab) may be an optimal choice to treat this formidable disease^23^. The ratio of spindle cells in tumors is also a known predictive factor of treatment outcome^16,21^. Patients with 25% or more spindle cells might have worse prognoses. In contrast, those with relatively small proportions of spindle cells might expect better clinical courses, even with VEGFR-TKI monotherapy^16^. However, of the three patients with less than 10% spindle cells in our study, two died within one year of initiation of systemic treatment. Consequently, patients with mRCC and poor IMDC risk scores or spindle features would be better treated with first-line ICI combination therapy even if they have contraindicated co-morbidities, such as autoimmune disorders or organ transplantations^24^.

In previous studies, several prognostic markers for axitinib treatment have been suggested, such as NLR^25^, serum albumin level^26^, serum lactate dehydrogenase level^26^, and serum CRP level^27^, among others. However, the model-development cohorts for these studies consisted of heterogeneous populations that included patients who had received a variety of treatments, including several VEGFR-TKIs. CRP value was related to poor PFS but not related to OS in our study. This might be caused by an interaction between NLR and CRP as an inflammatory representation, and both CRP and NLR were left over after the Cox hazard model. However, we still expect that CRP is a predictor of good prognosis in patients with mRCC^28,29^.

Our study is limited by its retrospective design, selection bias, unmeasured confounding factors, changes in clinical practice patterns over time, and the fact that the cohort was previously treated with VEGFR-TKI monotherapy before the immunotherapy era. Nevertheless, this study provides additional data that will help clinicians identify patients with mRCC who would receive long-term benefits from axitinib. Further studies on cohorts treated with immune-oncology therapies and longer follow-up times will be needed to validate our model.

## Conclusions

Our findings show that patients with mRCC and spindle histology or poor IMDC risk scores had worse OS and that high CRP levels predicted shorter PFS in first-line axitinib treatment. ICI combination therapies might be more suitable for initial management of such patients.

## Supplementary information

Supplementary Information.

## Data Availability

The datasets generated during and/or analyzed during the current study are available from the corresponding author on reasonable request.

## Abbreviations

ICI: Immune checkpoint inhibitors
CTLA-4: Cytotoxic T-lymphocyte associated antigen-4
mRCC: Metastatic renal cell carcinoma
VEGFR: Vascular endothelial growth factor receptor
TKI: Tyrosine kinase inhibitor
IMDC: International Metastatic Renal Cell Carcinoma Database Consortium
ECOG: Eastern Cooperative Oncology Group
PS: Performance status
ORR: Objective response rate
PFS: Progression-free survival
OS: Overall survival
AE: Adverse event
NLR: Neutrophil–lymphocyte ratio
CRP: C reactive protein
HR: Hazard ratio
CI: Confidence interval
